# Stochastic Model of the Adaptive Immune Response Predicts Disease Severity and Captures Enhanced Cross-Reactivity in Natural Dengue Infections

**DOI:** 10.3389/fimmu.2021.696755

**Published:** 2021-08-17

**Authors:** Hung D. Nguyen, Sidhartha Chaudhury, Adam T. Waickman, Heather Friberg, Jeffrey R. Currier, Anders Wallqvist

**Affiliations:** ^1^Biotechnology High Performance Computing (HPC) Software Applications Institute, Telemedicine and Advanced Technology Research Center, U.S. Army Medical Research and Development Command, Fort Detrick, MD, United States; ^2^Henry M. Jackson Foundation for the Advancement of Military Medicine, Bethesda, MD, United States; ^3^Center for Enabling Capabilities, Walter Reed Army Institute of Research, Silver Spring, MD, United States; ^4^Department of Microbiology and Immunology, State University of New York Upstate Medical University, Syracuse, NY, United States; ^5^Viral Diseases Branch, Walter Reed Army Institute of Research, Silver Spring, MD, United States

**Keywords:** dengue infection, affinity maturation, immune response, cross-reactive antibodies, humoral immunity, cellular immunity

## Abstract

The dengue virus circulates as four distinct serotypes, where a single serotype infection is typically asymptomatic and leads to acquired immunity against that serotype. However, the developed immunity to one serotype is thought to underlie the severe manifestation of the disease observed in subsequent infections from a different serotype. We developed a stochastic model of the adaptive immune response to dengue infections. We first delineated the mechanisms initiating and sustaining adaptive immune responses during primary infections. We then contrasted these immune responses during secondary infections of either a homotypic or heterotypic serotype to understand the role of pre-existing and reactivated immune pathways on disease severity. Comparison of non-symptomatic and severe cases from heterotypic infections demonstrated that overproduction of specific antibodies during primary infection induces an enhanced population of cross-reactive antibodies during secondary infection, ultimately leading to severe disease manifestations. In addition, the level of disease severity was found to correlate with immune response kinetics, which was dependent on beginning lymphocyte levels. Our results detail the contribution of specific lymphocytes and antibodies to immunity and memory recall that lead to either protective or pathological outcomes, allowing for the understanding and determination of mechanisms of protective immunity.

## Introduction

Dengue is the leading mosquito-borne viral illness in the world, infecting an estimated 390 million humans each year (1). The causative agent of the disease is the dengue virus (DENV), which belongs to the family of *Flaviviridae* in the genus *Flavivirus*. Humans can acquire symptomatic DENV infection multiple times owing to the circulation of four antigenically distinct serotypes (DENV-1 to DENV-4), which makes combating this disease difficult. A primary dengue infection is typically asymptomatic with only a minority of patients exhibiting a mild, uncomplicated dengue fever (DF). Immunization gained from the primary infection provides immune memory responses that are neutralizing and largely protective to viruses of the same serotype upon re-exposure during such a secondary homotypic infection. However, infection with a different serotype (i.e., secondary heterotypic infection) often results in severe disease manifestations, such as dengue hemorrhagic fever (DHF), and occasionally, death (2–4). This pattern of outcomes has led to the hypothesis that pre-existing immunity to dengue virus gained during a primary infection underlie an enhanced secondary heterotypic infection (5–7).

Simulations of the immune system using sophisticated models of the affinity maturation process are useful in providing a unifying framework for not only predicting disease severity of natural dengue infections, but also allowing us to ask non-trivial question of the origin and progress of dengue infections and delineate the immune response in granular detail. However, simulation studies on immune response to dengue infection have only emerged recently, using a variety of simplified models of the immune system (8–13). Moreover, only a few models have been developed to study within-host dengue viral dynamics (11) and no single study has explicitly represented multiple serotypes and epitopes, which are key requirements to model multiple infections by different serotypes.

In this study, we extended the affinity-maturation framework developed by Chaudhury et al. for malaria infections (14) to model dengue infections with explicit representation of multiple serotypes and epitopes. We made two major additions to account for the presence of T cells and viral replication processes. The first addition allowed us to model the interactions between B cells and helper CD4^+^ T cells in response to the invasion by a dengue virus to the germinal center (GC). The second addition enabled us to monitor viremia dynamics as the dengue virus undergoes replication during its growth period and its clearance by either antibodies or cytotoxic CD8^+^ T cells. Together, these additions enabled us to model the T-dependent (TD) immune response to the presence of dengue virus. This response lasts for weeks and involves co-stimulation of B cells and helper CD4^+^ T cells within the germinal center of a host lymph node. During affinity maturation, these B cells undergo proliferation, somatic hypermutation, and differentiation. These additions permitted us to capture the hallmark feature of the TD immune response that is responsible for the production of high-affinity antibodies with numerous germline mutations and the formation of long-lived plasma cells and memory B cells. Moreover, this model enabled us to stochastically simulate affinity maturation and contrast the mechanisms initiating and sustaining adaptive immune responses during primary infection with the immune pathways that are pre-existing and reactivated during secondary infection. This permitted us to determine the contributions of naïve B cells, CD4^+^ T cells, CD8^+^ T cells, and antibodies to immunity and memory recall that may lead to protective or pathological outcomes. Here, our objective is to employ this model to stochastically simulate affinity maturation occurring in natural infections using a large population sample to capture the range of possible immune responses. This will allow us to model and fully characterize the immune parameters in the model for both typical and rare responses and map disease severity to these parameters.

Because our theoretical model cannot predict clinical symptoms, we have to rely on physical evidences such as viremia, antibody serum, and activated B and T cells to identify patients with a certain disease manifestation, ranging from non-symptomatic to symptomatic cases that in turn can be classified as either mild or severe. We hypothesize that the immune responses to natural dengue infections carry latent information that can be predictive of the disease severity of secondary infection. We applied our model to simulate homotypic and heterotypic natural infections in order examine how the antibody response changes between primary and secondary infections. We were interested in determining aspects of the antibody response that are responsible for protection and investigating mechanisms underlying the shift from a protective primary antibody response to a non-protective secondary antibody response. Understanding the immune responses associated with homotypic and heterotypic natural infections provide the foundation for rationally develop and optimize vaccine design, delivery, and minimize the risk of severe adverse effects associated with DHF.

## Methods

### Stochastic Model of Affinity Maturation

**Figure 1:**
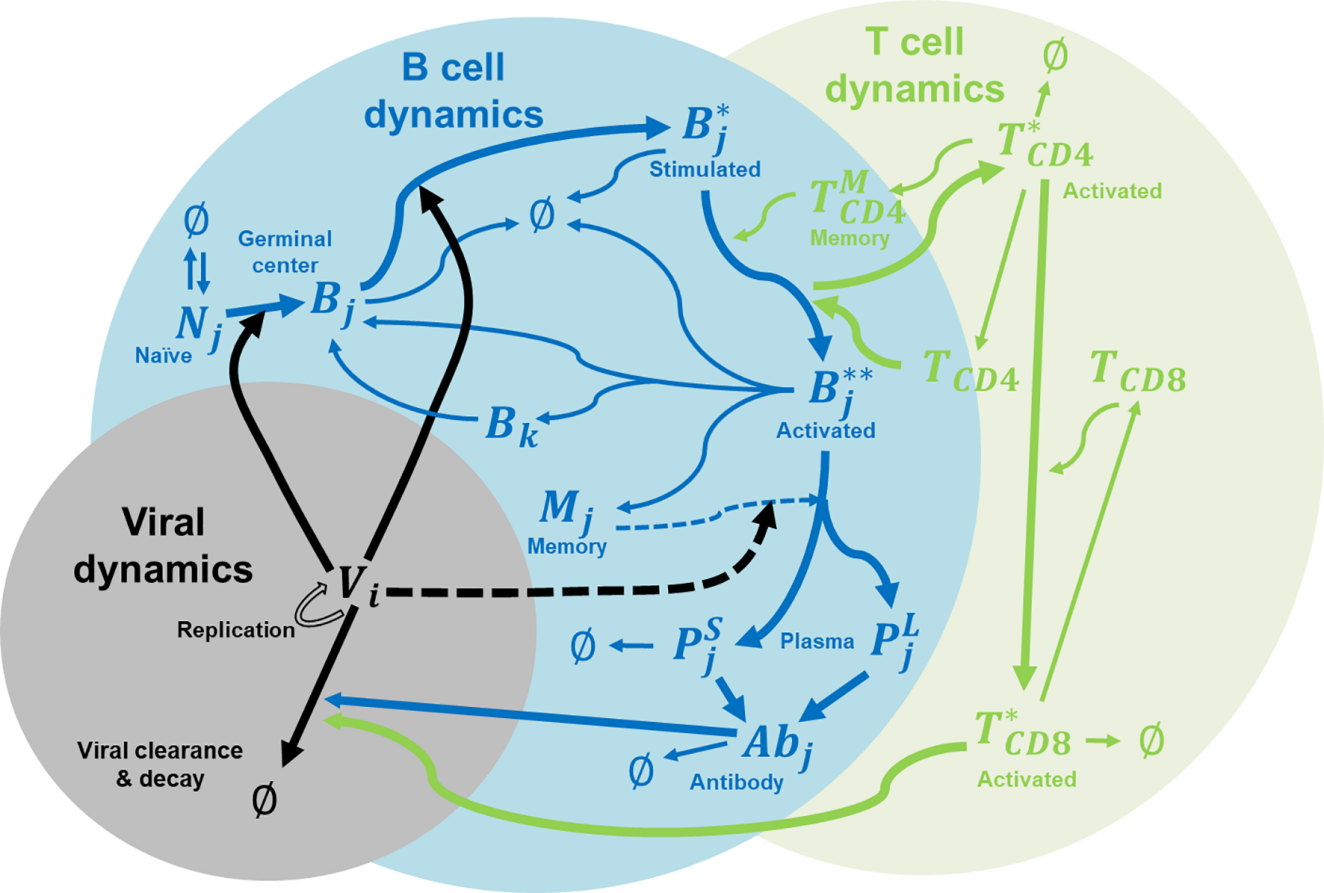
Immune system model for affinity maturation. A summary of the immune system model that is used to simulate affinity maturation, including B and T cell stimulation, proliferation, mutation, differentiation, Ab production, and virus replication and clearance. The components in the model include naïve B cells (*N*), germinal center B cells (*B*), stimulated B cells (B*), activated B cells (B**), memory cells (*M*), plasma cells (*P* as short-lived, *S*, and long-lived, *L*), virus (V), CD4^+^ T cells (*T_CD4_*), activated CD4^+^ T cells (T*_CD4_), memory CD4^+^ T cells $\left(T_{CD4}^{M}\right)$, CD8^+^ T cells (*T_CD8_*), activated CD8^+^ T cells (T*_CD8_), and Abs. Subscript *i* denotes epitope genotype, *j* denotes paratope genotype, and *k* denotes mutated paratope genotype.

In this study, we extended the affinity-maturation model developed by Chaudhury et al. (14) for malaria infections to model dengue infections. **Figure 1** shows the major components of the coupled viral, B cell, and T cell dynamics that accounts for affinity maturations, B and T cell stimulations, proliferation, mutation, differentiation, Ab production, viral replication and virus clearance. The model allows for the interactions between B cells and helper CD4^+^ T cells in the germinal center in response to the viral infection. The incorporation of viral dynamics allow us to monitor viremia as the dengue virus undergoes replication during its growth period and its clearance by either antibodies or cytotoxic CD8^+^ T cells.

We modeled B cell affinity maturation as a set of rate reactions, similar to chemical reactions, which describe the underlying immunological processes, such as virus binding, B-cell activation T cells, B cell replication, etc. We carried out stochastic simulations of affinity maturation by applying the Gillespie algorithm (15) as adapted by Woo and Reifman for modeling the immune system (16) to this set of rate equations outlined below. We coded the algorithm in Python, and the source code is freely available on GitHub.

### Immunological Shape Space

We used the immunological shape space model developed by Smith et al. (17) to model multiple epitopes for multiple serotypes of dengue virus. This allowed us to describe the antigenic relationships between each serotype, at the epitope level, with respect to B cell specificity and cross-reactivity. Details of the immunological shape space model are given in earlier papers (14, 16).

### Immune System Components

In this model, each virus (denoted as *V*) belonging to one of the four dengue serotypes (DENV-1, DENV-2, DENV-3, and DENV-4) has its own genotype denoted by the subscript *i*. Each virus serotype contains the epitope genotypes for four epitopes: PrM from the pre-membrane protein, fusion loop (FL), domain III (DIII), and hinge from the envelope protein. All sequences are listed in **Table S1** and their parameters of immunogenicity and clearance are listed in **Table S2**. These parameters were taken from a previous study by Chaudhury et al. (18) who carried out a large-scale analysis of 400 unique monoclonal dengue-specific antibodies to map each epitope at the domain level with its antigenic activity. Each epitope (and paratope) is symbolically represented as a 20-character string made up of four unique characters. The sequence difference between all four serotypes of each epitope shows that the antigenic distance is 0 for the PrM epitope, 1 for FL, 4 for DIII, and 5 for hinge.

All B cells and Abs in the system are likewise denoted by a paratope genotype (subscript *j*), which is also represented as a 20-character string made up of four unique characters. There are seven types of B cells: naïve (*N*), germinal center GC (*B*), stimulated (B^*^), activated (B^**^), short-lived plasma (*P^S^*), long-lived plasma (*P^L^*), and memory (*M*), along with antibodies (*Ab*), all of which have paratope genotypes. There are five types of T cells: helper CD4^+^ (*T_CD4_*), activated CD4^+^ (T*_CD4_), memory CD4^+^ $\left(T_{CD4}^{M}\right)$, cytotoxic CD8^+^ (*T_CD8_*), and activated CD8^+^ (T*_CD8_). In this version of our model, all B cells and Abs can be identified as either DENV specific or cross-reactive; however, all T cells are described as an overall abundance of polyclonal CD4^+^ and CD8^+^ T cells. These major components of B and T cells are included in this version of our modeling because they are being either stimulated by dengue virus or capable of neutralizing dengue virus. For simplicity, other subtypes of B and T cells such as TFH or NKT cells and other immune cells such as dendritic cells and macrophages are not considered in this model despite the fact they are known as being infected by dengue virus and play a relevant role in immune response.

Subscript *i* denotes epitope genotype of viruses while subscript *j* denotes paratope genotype of B cell receptors and antibodies. Cross-reactivity of an antibody to two virus serotypes happens when the paratope genotype of an antibody has non-zero binding energy with epitope genotype of both virus serotypes (as having the number of mismatches between every two sequences of seven or fewer).

### Rate Equations

We modeled affinity maturation using a set of equations that describe B cell stimulation and proliferation with the aid of helper CD4^+^ T cells, memory and plasma cell differentiation, Ab production, and virus clearance by either Abs or cytotoxic CD8^+^ T cells. In the system, the genotype of every virus epitope or paratope of B cell receptor and antibody was explicitly described (by either a subscript *i* or *j* in the following equations). All parameters used below are summarized in **Table S2**. The rate constant parameters for all reactions related to B cells were taken from a previous study by Chaudhury et al. (18), which provided a detailed description of those parameters. However, the rate constant parameters for T cells were tuned in this study to capture certain key features of T cell response as discussed in the Results section.

Dengue virus is known to undergo rapid viral growth in infected patients over a period of 14 days, reaching a maximum of 10^6^ to 10^10^ units/ml (19–21). This replication process is modeled as a first-order reaction (Eq. 1a) forming two copies from every dengue virus with a rate constant *k_V_*. Moreover, there is an intrinsic decay rate (*g_V_*) of dengue virus due to non-specific clearance processes in the body (Eq. 1b). It is related to the half-life of dengue virus, which is 5.2 hours at 37°C (22, 23). Each virus (denoted as *V*) belonging to one of the four dengue serotypes (DENV-1, DENV-2, DENV-3, and DENV-4) has its own genotype denoted by the subscript *i*.

(1a) $$\;V_{i}\underset{k_{V}}{\to }2V_{i}$$

(1b) $$\;V_{i}\;\underset{g_{V}}{\to }0$$

In this model, there was a revolving concentration of naïve B cells (*N*) that was continuously replenished and whose formation and decay were described as zero-order (Eq. 2a) and first-order (Eq. 2b) reactions, respectively. This *N* concentration was randomly chosen for every simulation, unlike the original model by Chaudhury et al. (14) in which *N* was fixed at the same value for all simulations, from a non-normal distribution whose median, minimum and maximum values were from a healthy population of 6-12 years old children (24). The B cell formation rate (*k_N_*) was set such that a steady-state concentration of naïve B cells was maintained for the given decay rate. That decay rate was set with a rate constant *g_N_* based on an estimated naïve B cell half-life of 4.5 d (25, 26) (Eq. 2b). In this case, any newly formed B cell was assigned a random paratope genotype (denoted as *j*).

(2a) $$0\underset{k_{N}}{\to }\;N_{j}$$

(2b) $$\;N_{j}\;\underset{g_{N}}{\to }0$$

The original model by Chaudhury et al. (14) used only naïve B cells with a nonzero binding affinity to any epitope in the system. Specifically, it limited naïve B cells to paratope genotypes with a minimum Hamming distance of 7 to any epitope. In contrast, our current model allows random naïve B cells whose paratope genotypes range from 7 to higher Hamming distances. In fact, only approximately 9% of naïve B cells with a nonzero binding affinity given by the Hamming distance of 7 while the rest of naïve B cells have zero binding affinity with the Hamming distance greater than 7. This allows naïve B cells to undergo a complete maturation process from zero to weak and then to strong binding affinity to any epitope in the system.

We modeled stimulation and migration of naïve B cells into GC B cells and stimulation of GC B cells into stimulated B cells (denoted as B*) following virus binding (Eq. 3a, 3b) as described in the original model (14).

(3a) $$N_{j}+\;V_{i}\;\underset{\sigma_{N}\gamma_{i}Qij}{\to }B_{j}+\;V_{i}$$

(3b) $$B_{j}+\;V_{i}\;\underset{\sigma_{N}\gamma_{i}Qij}{\to }\;B_{j}^{*}+\;V_{i}$$

Similar to naïve B cells, we modeled the formation and decay of helper CD4^+^ T cells (denoted as *T_CD4_*) as zero-order (Eq. 4a) and first-order (Eq. 4b) reactions, respectively. Also, the initial *T_CD4_*concentration was randomly chosen for every simulation to model a healthy population of 6-12 years old children (24). The CD4^+^ T cell formation rate (*k_T1_*) was set such that a steady-state concentration of CD4^+^ T cells was maintained for the given decay rate *k_T2_.*

(4a) $$0\underset{k_{T1}}{\to }T_{CD4}$$

(4b) $$T_{CD4}\underset{k_{T2}}{\to }0$$

We modeled activation and migration of stimulated B cells into activated B cells (denoted as B**) following a binding with *T_CD4_* (Eq. 5) as a second-order reaction with a rate constant *k_T5_*. *T_CD4_*is also undergoing activation to form T*_CD4_.

(5) $$B_{j}^{*}+T_{CD4}\;\underset{k_{T5}}{\to }\;B_{j}^{\text{**}}+T_{CD4}^{*}$$

At this point, activated B cells are ready to undergo proliferation (Eq. 6a), differentiation into memory (Eq. 6b) and plasma (Eq. 6c) cells, and decay (Eq. 6d) as described in the original model (14). The major difference in this version compared to the original model (14) is that plasma cells are split into short-lived and long-lived plasma cells (denoted as *P^S^* and *P^L^*, respectively) with an unequal probability α (Eq. 6c) tilted favorably towards short-lived plasma cells.

(6a) $$B_{j}^{\text{**}}\;\underset{rR_{jk}}{\to }\;B_{j}+B_{k}$$

(6b) $$B_{j}^{\text{**}}\;\underset{\delta }{\to }\;M_{j}$$

(6c) $$B_{j}^{\text{** }}\underset{\delta }{\to }\alpha P_{j}^{S\;}+\text{ (1- }\alpha )P_{j}^{L\;}$$

(6d) $$B_{j}^{\text{** }}\underset{\text{max}(\eta ,g_{B})}{\to }0$$

Serum Abs are produced from short-lived and long-lived plasma cells (Eq. 7a and 7b) with an equal probability. However, only short-lived plasma cells undergo decay (Eq. 8) since long-lived plasma cells are known to last for years and maintain the capability to produce Abs (27, 28).

(7a) $$P_{j}^{S\;}\underset{k_{Ab}}{\to }P_{j}^{S\;}+\;Ab_{j}$$

(7b) $$P_{j}^{L\;}\underset{k_{Ab}}{\to }P_{j}^{L\;}+\;Ab_{j}$$

(8) $$P_{j}^{S\;}\underset{g_{P}}{\to }0$$

Memory cells become plasma cells when stimulated by the presence of virus (Eq. 9), and play an important role in providing an immediate Ab response without going through an affinity maturation process starting from naïve B cells. Moreover, they are estimated to have lifespans of months to years (29) and, hence, there is no decay of this population in the simulation.

(9) $$M_{j}+\;V_{i}\underset{\sigma_{M}\gamma_{i}Q_{ij}}{\to }P_{j}^{S\;}+P_{j}^{L\;}+V_{i}$$

Abs bind to a virus in order to clear it through a second-order reaction (Eq. 10a). They decay with a rate *g_Ab_* based on a half-life of 10 d (Eq. 10b) as described in the original model (14).

(10a) $$Ab_{j}+\;V_{i}\underset{\rho_{i}Q_{ij}}{\to }\;Ab_{j}$$

(10b) $$Ab_{j\;}\underset{g_{Ab}}{\to }0$$

Similar to activated B cells, T*_CD4_ undergo differentiation into either original *T_CD4_* (Eq. 11a) or memory *T_CD4_* (Eq. 11b) and decay (Eq. 11c) as first-order reactions with rate constants *k_T6_*, *k_T7_*, and *k_T8_*, respectively

(11a) $$T_{CD4}^{*}\;\underset{k_{T6}}{\to }T_{CD4}$$

(11b) $$T_{CD4\;}^{*}\underset{k_{T7}}{\to }T_{CD4\;}^{M}$$

(11c) $$T_{CD4\;}^{*}\underset{k_{T8}}{\to }\;0$$

Memory *T_CD4_* cells can be reverted back to activated *T_CD4_* cells through a second-order reaction with rate constant *k_T9_* (Eq. 12) and they have lifespans of 2-3 years (30) and are not tagged for decay in the simulation.

(12) $$B_{j}^{*}+T_{CD4\;}^{M}\;\underset{k_{T9}}{\to }\;B_{j}^{\text{**}}+T_{CD4}^{*}$$

Similar to *T_CD4_* cells, we modeled the formation and decay of cytotoxic CD8^+^ T cells (denoted as *T_CD8_*) as zero-order (Eq. 13a) and first-order (Eq. 13b) reactions, respectively. The CD8^+^ T cell formation rate (*k_T3_*) was set such that a steady-state concentration of CD8^+^ T cells was maintained for the given decay rate *k_T4._*Also, the initial *T_CD8_*concentration was randomly chosen for every simulation from a non-normal distribution of a healthy population of 6-12 years old children (24).

(13a) $$0\underset{k_{T3}}{\to }T_{CD8}$$

(13b) $$T_{CD8}\underset{k_{T4}}{\to }0$$

Cytotoxic CD8^+^ T cells have to be activated by T*_CD4_into T*_CD8_(Eq. 14) through a second-order reaction with rate constant *k_T10_*.

(14) $$T_{CD4}^{*}+T_{CD8}\;\underset{k_{T10}}{\to }T_{CD4}^{*}+T_{CD8}^{*}$$

Once activated, cytotoxic CD8^+^ T cells can clear virus through a second-order reaction with rate constant *k_T11_* (Eq. 15)

(15) $$T_{CD8}^{*}+\;V_{i}\underset{k_{T11}}{\to }T_{CD8}^{*}$$

Activated CD8^+^ T cells can also be reverted back to original *T_CD8_*cells (Eq. 16a) or undergo decay (Eq. 16b) through first-order reactions with rate constants *k_T12_* and *k_T13,_*respectively.

(16a) $$T_{CD8}^{*}\;\underset{k_{T12}}{\to }T_{CD8}$$

(16b) $$T_{CD8\;}^{*}\underset{k_{T13}}{\to }\;0$$

These rate constants are given in **Table S2**

### Simulation Conditions

The simulation conditions were set up to reflect natural dengue infections observed in the clinic (31). Three sets of simulations were performed for each heterotypic and homotypic infection, each set constructed from 10,000 independent simulations (**Figure S1**). For the heterotypic set, a dose at a concentration of 10^5^ copies/ml of DENV-1 was inserted into the system at day 0, another dose at the same concentration of DENV-2 was inserted into the system exactly a year later. For the two homotypic sets, a dose at a concentration of 10^5^ copies/ml of either DENV-1 or DENV-2 was inserted into the system at day 0, another dose at the same concentration of either DENV-1 or DENV-2 was inserted into the system exactly a year later. Each virus was represented as four epitopes: PrM, FL, DIII, and hinge (**Table S1**).

In this stochastic model, each simulation produced a unique trajectory that represented the outcome of a single individual. At the beginning of each simulation, all variables (concentrations of B and T cells) were set at zero except for the concentrations of naïve B cells (*N*), helper CD4^+^ T cells (*T_CD4_*) and cytotoxic CD8^+^ T cells (*T_CD8_*). Those concentrations were randomly chosen from respective non-normal distributions whose median, minimum and maximum values were from a healthy population of children of 6-12 years old (24).

We carried out 10,000 independent simulation for set of heterotypic and homotypic infections. Each simulation was run for two years, one year for each infection, and lasted ∼1 hour on a desktop computer. We report the average values with error bars denoting one standard deviation at each time point, as well as the overall distribution of outcomes.

## Results

### Model Captures Viral Growth Kinetics

**Figure 2:**
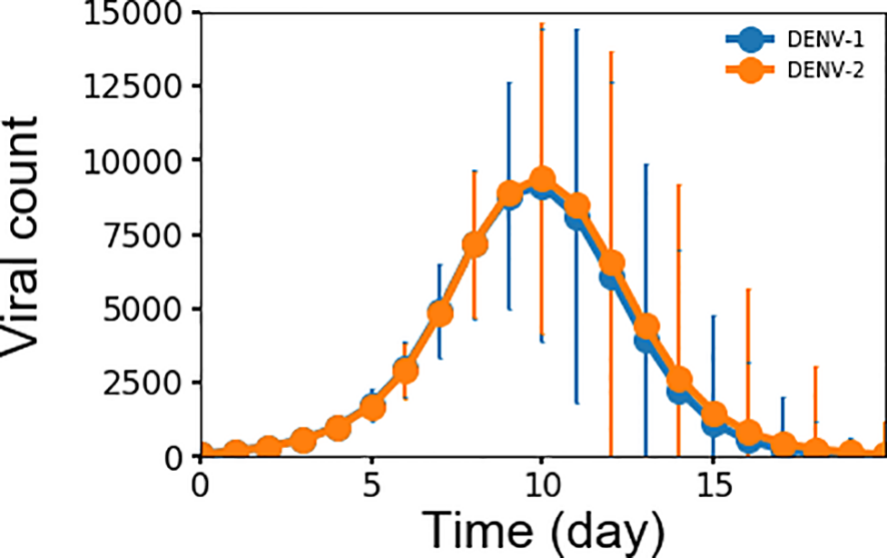
Averaged concentration (n units x 10^3^/ml) of virus particles as a function of time during primary infections for both DENV-1 and DENV-2 from 10,000 simulations, error bars are shown as the standard deviation.

Monitoring viremia is a key factor in detecting a primary dengue infection. Dengue infection begins with a mosquito bite, which initiates an incubation period of 4–6 days before viremia shows in significant numbers. Peak viremia causes high fever in some patients and coincides with the launching of antibody production, which promotes clearance of virus infection. Although viremia may be subsiding, a small number of patients may still experience hemorrhaging and/or shock that is thought to be immune-mediated (7, 32). **Figure 2** shows the simulated average concentration of DENV-1 and DENV-2 as a function of time during primary infections that correspond to the complete viremia kinetics. Since the replication rate is the same for all virus serotypes in this model, there is no differences between the concentrations for DENV-1 and DENV-2. There was a period of lag time for four days when the virus grew slowly, corresponding to the incubation period as seen in clinical data (19, 33), then the virus population quickly increased, reaching a peak concentration at approximately 10^7^ units/ml on day 10, at which viremia started to undergo clearance with the viral count decreasing to zero on day 16. This is in agreement with published results from clinical and laboratory investigations (19, 33) that showed mostly the secondary half of viremia starting from peak viremia and the descent from peak viremia to ~1000 units/ml, which is the assay limit of virus detection of RT-PCR signal, takes 4-6 days (19). Peak viremia from our model of 10^7^ units/ml is on the lower end of the reported concentrations that range between 10^6^ to 10^10^ units/ml (19–21). However, our model results represent the average peak concentration of all subjects of which the majority is non-symptomatic and only a minority would be symptomatic with DF or DHF, whereas the values of peak viremia from experimental data were only reported for patients with DF or DHF and most likely skewing these numbers to a higher range of viremia.

### Model Captures Key Features of Affinity Maturation and Immune Response

**Figure 3:**
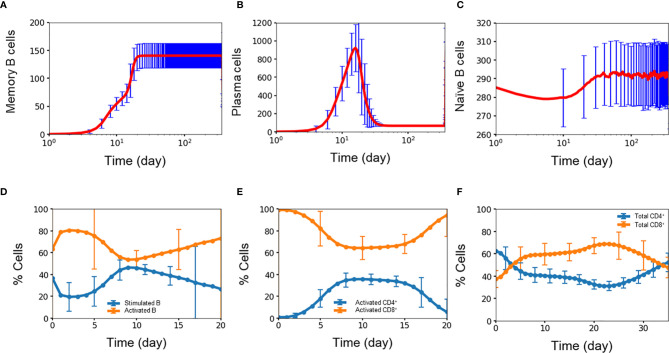
Concentration (n units x 10^3^/ml) of **(A)** memory B cell, **(B)** plasma B cell, **(C)** naïve B cell, percentage of **(D)** stimulated B cell and activated B cell, **(E)** CD4^+^ T cell and CD8^+^ T cell, **(F)** activated CD4^+^ T cell and activated CD8^+^ T cell concentration as a function of time during primary infections. Average values from 10,000 independent simulations are shown, error bars are shown as the standard deviation.

Experimental studies show that B cells dynamics exhibit three signature trends during dengue infection with respect to the amount of memory B cells, plasma B cells, and naïve B cells present during the first week of infection and compared with four months after infection (34). **Figures 3A–C** show the corresponding time courses for the simulated B cell populations over one year. First, the amount of memory B cells increases over time due to their long lifetimes and lack of decay (29). **Figure 3A** shows the simulated memory B cell population exhibiting an initial rapid growth during the initial two weeks after which the population plateaus out to a large value and remains stable during the simulation. In comparison, the amount of memory B cells is seven times greater at month 4 than compared with the average value of day 4-7 (*p*-value < 0.0001). Second, plasma B cells consist of both short-lived and long-lived cells. Initially during the first two weeks (34) the number of both short-lived and long-lived cells increases when the virus is present, after which the short-lived cells start to decay while the long-lived cells remain. Because short-lived cells are generated at a greater amount than long-lived cells, the fall-off in the total plasma cell population is significant. **Figure 3B** show the corresponding behavior of the simulated plasma cell population with an initial rapid increase and subsequent steep decay. In comparison, the total amount of plasma cell at month 4 was twice as low as the average value of day 4-7 (*p*-value < 0.03). Finally, naïve B cells are converted into germinal center B cells during the first two weeks of any infection requiring that after each infection, naïve B cells are replenished to their original concentrations at equilibrium. **Figure 3C** shows this signature depletion and restoration of the naïve B cell population as captured in the simulation. The resulting amount of naïve B cells was only 3.5% greater at month 4 than that during the first week (*p*-value < 0.02). This shows that even though only a small population of naïve B cells was converted into germinal center B cells, that amount was enough to mount a strong defense against the dengue virus. Since the formation rate of naïve B cells relatively high, naïve B cells are replenished quickly due to the fact this population of young children in this study have lymphocyte numbers higher than those of older populations.

Activation of B cells and T cells is important in the overall immune response during an infection (35). **Figures 3D–F** show the average simulation results for these cell populations during the initial period post infection. For B cells, rapid conversion of stimulated B cells into activated B cells is imperative for providing protections against the infection. A high amount of activated B cells ensures that there is a sufficient capacity available for further differentiation into plasma cells that produce antibodies for immediate protection as well as memory B cells for long-term protection. **Figure 3D** shows the coupled dynamics of stimulated and activated B cells during the initial three weeks with a rapid conversion of stimulated B cells into active B cells within one day, the build-up of the stimulated population within a week, and the long-term decay of this population as the infection is slowly cleared.

Activated CD4^+^ cells and CD8^+^ cells play important, but different roles in the immune response. The role of CD4^+^ cells is to activate both B cells and CD8^+^ cells so CD4^+^ cells are constantly generated and consumed during each activation event. On the other hand, the role of CD8^+^ cells is to stay around in high numbers for the purpose of clearing viruses, which is critical during the first period of an infection when production of antibodies is delayed. **Figures 3E, F** shows this behavior among the simulated population of T cells, i.e., activated CD8^+^ cells were generated at a disproportionally higher amount than that of active CD4^+^ cells during the week of infection and at a stable yet higher amount of 1.5 times (*p*-value <0.00001) during the second week of infection, even though the initial amount of CD4^+^ T cells was almost double of the amount of CD8^+^ T cells. This qualitatively agrees with the results from a study using flow cytometry by Mathew and coworkers who found significant expansion and activation of peripheral follicular helper T cells during acute dengue virus infection from Thai children (36). In particular, they found an increase in activated CD8^+^ frequencies coincident with a decrease in activated CD4^+^ frequencies during acute infection, giving a higher amount of the total CD8^+^ cells compared to the total amount of CD4^+^ cells.

### Serotype-Dependent Viral Growth During Secondary Infection: More Viral Replication During Heterotypic Infection Than Homotypic Infection

**Figure 4:**
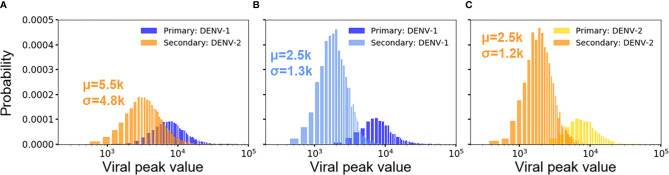
Distribution of viral peaks from primary and secondary infections in heterotypic **(A)** and homotypic infections **(B, C)** from 10,000 independent simulations. Each viral peak value of concentration (n units x 10^3^/ml) comes from one simulation. Each plot includes the average concentration of viral peak (μ) and standard deviation (σ) for comparison.

For both heterotypic and homotypic infections, the primary infections results in subsequent lower viral growth in the secondary infections, observed both in clinical observations and experiments (19, 20, 37). **Figures 4A–C** show the distribution of peak viral counts (peak viremia) for both heterotypic and homotypic infections based on 10,000 simulations, each lasting for two years. For all secondary infections, the peak viremia was significantly reduced compared to the primary infections. However, the magnitude of the change was distinct between homotypic and heterotypic infections, with the heterotypic infection showing the least reduction in peak viremia values from 12.3·10^3^ to 5.5·10^3^ (**Figure 4A**) compared to a reduction from 12.3·10^3^ to 2.5·10^3^ for secondary homotypic infections (**Figures 4B, C**), (*p*-value <0.00001). The standard deviation of the peak viremia values was four times as broad (4.8·10^3^) for secondary heterotypic infections compared to the secondary homotypic infections (1.2-1.3·10^3^) Although the stochastic simulations show an extensive variability in individual immune responses, the overall peak viremia difference of the hetero- *vs.* homotypic secondary infections show that the differences in response arise due to serotype heterogeneity, rather than from the stochastic nature of simulations.

### Key Differences Between Primary and Secondary Heterotypic Infections on Immune Response

**Figure 5:**
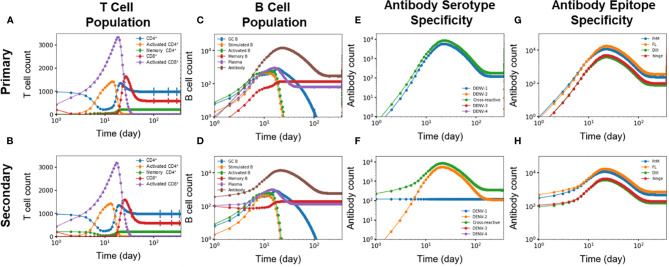
**(A, B)** T cell population (n units x 10^3^/ml), **(C, D)** B cell population, antibody specificity at **(E, F)** serotype and **(G, H)** epitope levels from primary and secondary heterotypic infections as a function of time from 10,000 independent simulations.

We identified five key differences of the immune response between primary and secondary heterotypic infections. **Figures 5A–H** summarizes these time-dependent differences and highlights changes in the immediate immune response as well as for the long-term protection. First, **Figures 5A, B** show how memory CD4^+^ T cells generated from primary infections are retained for the secondary infections and then differentiated into activated CD4^+^ T cells (**Figure 1**). Indeed, these memory CD4^+^ T cells started from zero in the beginning of primary infections whereas they started at a non-zero value at the beginning of secondary infections. Second, **Figures 5C, D** show antibodies, memory B cells, and plasma B cells being generated during primary infections starting from zero and are retained in response to the secondary infections (as shown as starting from non-zero values at the beginning of secondary infections). Third, **Figures 5E, F** show DENV-1-specific antibodies being generated during primary infections, retained, and maintained intact during secondary infections, while DENV-2-specific antibodies were only generated during the secondary infections. Fourth, **Figures 5E, F** also show that cross-reactive antibodies were generated during primary infections, retained, and then regenerated during secondary infections. Finally, **Figures 5G, H** show generation of epitope-specific antibodies being generated during primary infections, retained, and then regenerated during secondary infections, even though both PrM-specific and FL-specific antibodies are generated at higher concentrations than DIII-specific and hinge-specific antibodies in both primary (**Figure 5G**) and secondary (**Figure 5H**) infections. Note that T cells are shown in **Figure 5** as an overall abundance of polyclonal CD4^+^ and CD8 T^+^ cells rather than DENV specific types.

In addition, **Figure S2** show another difference in the immune response between primary and secondary heterotypic infections as related to the affinity of GC B cells, memory B cells, plasma cells, and antibodies. Production of antibodies that are of high affinity was delayed for three days for medium-affinity antibodies to almost a week for maximum-affinity antibodies during primary infections (**Figure S2G**). In contrast, production of antibodies of any affinity level was immediate, starting on day one during secondary infections (**Figure S2H**).

### Disease Classification by Secondary Viremia Peak Shows Good Agreement With Clinical Observations

Secondary dengue infections are manifested as either non-symptomatic or symptomatic cases, with the latter classified as either moderate or severe that may require hospitalization. In a comprehensive study, Salje et al. identified 65% of the infected patient population of 3,451 children in Thailand as non-symptomatic (31). Of the 35% symptomatic children, almost a third (~10% of the total infected children) required hospitalization, half of which (~5% of the total infected children) exhibited symptoms associated with DHF. In other words, they classified infected children into four categories of disease severity: non-symptomatic (65%); symptomatic without hospitalization (25%); symptomatic, hospitalized yet without DHF (5%); and symptomatic, hospitalized with DHF (5%). We used this classification scheme to label four categories of disease severity as non-symptomatic, mild, moderate, or severe.

While a clinical study can classify infected patients by observing their symptoms, our modeling study has to rely on other benchmarks. Fortunately, Vaughn et al. demonstrated that peaked viral titers of patients with secondary infections correlate with three different disease categories: DF, DHF grades 1/2, and DHF grade 3 (20). However, because the sample size of their study is relatively small, consisting of just 74 patients, the maximum viral titer values for these disease categories might not represent a clean separation for a larger population. To address this, we employed a machine learning technique of k-means clustering to separate the population based on their secondary viral peak values, which are correlated with the initial concentration of naïve B cells (**Figure S3A**). This technique allowed us to separate the population into four separate clusters (**Figure S3A**) that correspond to four different levels of disease severity: non-symptomatic, mild, moderate, and severe. For both secondary viral peak maximum and concentration of naïve B cells, the centroids of these four clusters presented a clear and significant separation of disease severity based on their *p*-values (<0.05). This method of correlation disease severity with the secondary viral titers is also supported by a recent systematic review of 30 studies with 3,316 patients, which revealed that viremia is significantly higher in DHF patients than those in DF patients in days 5 to 6 when peaked viremia were reached (38).

Disease classification of heterotypic infections predicted by our model as shown in **Table 1** is in agreement with the results from the clinical observation of Salje et al. (31). Moreover, disease classification of homotypic infections predicted by our model as shown in **Table 1** is also in agreement with the clinical observation of Waggoner et al. (39) as explained in more detail in the Discussion section.

**Table 1:** Average percentages of patients sorted by disease severity from both simulation (heterotypic and homotypic) and clinical studies.

Data Source	Model Simulations		Clinical Observations (31)
Infection type	Homotypic	Heterotypic	Heterotypic
% non-symptomatic	97.58	66.52	65
% symptomatic	2.42	33.48	35
*degree of severity among symptomatic cases*			
% mild	2.32	26.53	25
% moderate	0.10	6.06	5
% severe	0.00	0.89	5

### Profile of Antibody Specificity and Cross-Reactivity Offers Underlying Clues for Disease Severity

**Figure 6:**
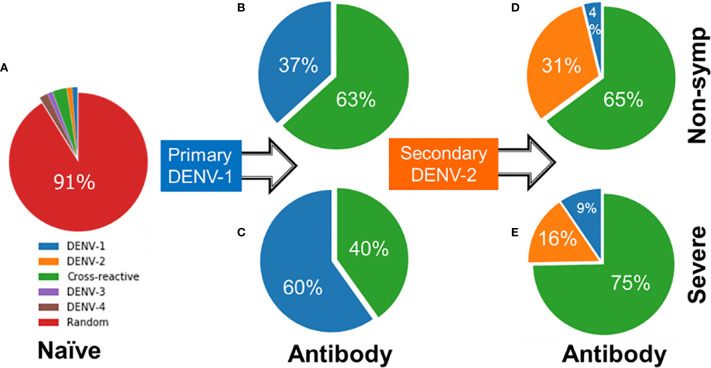
Starting from **(A)** naïve B cells, profile of antibodies generated during **(B, C)** primary infections by DENV-1 and **(D, E)** secondary infections by DENV-2 showing percentages of specific, cross-reactive or antibodies of no binding affinity to any dengue serotype (random) for both severe and non-symptomatic cases.

**Figures 6A–C** shows contrasting antibody profiles for non-symptomatic and severe cases. Initially, the large majority of naïve B cells have no affinity for any virus epitope as indicated by 91% of the naïve B cell population having random genotypes (**Figure 6A**). As B cells of non-symptomatic cases undergo affinity maturation during a primary infection by DENV-1, they produce antibodies that are overwhelmingly cross-reactive (63%) as compared to DENV-1-specific (37% in **Figure 6B**). In contrast, the B cells associated with severe disease cases during a primary infection produce antibodies that are mainly type-specific for DENV-1 (60%) compared to cross-reactive (40% in **Figure 6C**). When subsequently infected with a heterologous DENV-2 serotype, the B cells of the non-symptomatic cases produced a similar majority of cross-reactive antibodies (65% in **Figure 6D**) as in the primary infections and a minority of type-specific antibodies for DENV-2 (31%); in addition a small amount of type-specific DENV-1 antibodies were also generated (4%). In contrast, B cells of severe secondary infections produced a lower percentage of type-specific antibodies for DENV-2 (16% **Figure 6E**), while retaining a higher percentage type-specific antibodies for DENV-1 (9%) and generating a large majority cross-reactive antibodies (75%). Lower amounts of type-specific antibodies for DENV-2 and higher amounts of cross-reactive antibodies among the severe cases allow the virus to escape clearance, thus permitting the virus to grow in larger numbers compared to the non-symptomatic cases.

The set of model simulation also allowed us to examine the evolved immune status prior to the secondary heterotypic DENV-2 infection. **Figures S4A, B** shows the profile of mature B cells, including memory B cells, plasma cells, antibodies, and a fresh line of naïve B cells, for non-symptomatic and sever cases. The percentage of DENV-1-specific mature and naïve B cell types was 22% for non-symptomatic cases compared to 37% for severe cases. When infected with DENV-2, some of these DENV-1-specific cells developed affinity for DENV-2, converting into cross-reactive antibodies while the remaining cells continue to produce DENV-1-specific antibodies. The source of the DENV-2-specific antibodies comes from non-serotype specific cells (designated as random cells). The lower percentage of random cells among the severe cases (37% in **Figure S4B**) produced fewer type-specific antibodies for DENV-2 (**Figure 6E**) compared to non-symptomatic cases (41% random cells in **Figure S4A**). Although the 4% difference is relatively small, the difference is sufficient to produce significantly more type-specific antibodies for DENV-2 of 31% for non-symptomatic cases (**Figure 6D**) compared to 16% for severe cases (**Figure 6E**) based on their *p*-values (<0.01).

### Correlate of Disease Severity to Lymphocyte-Dependent Immune Response

**Figure 7:**
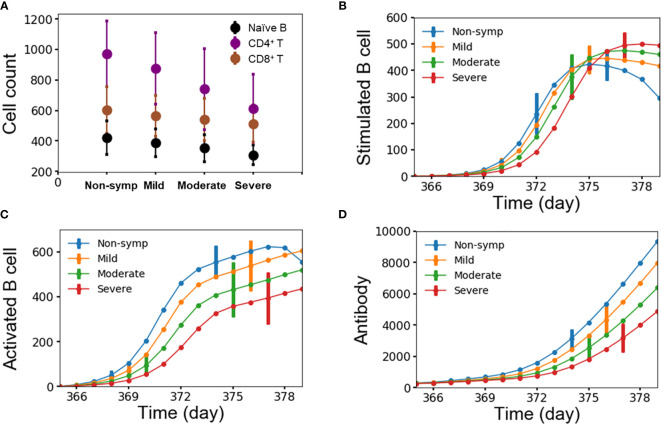
**(A)** Average number of lymphocytes for each group of disease severity; **(B–D)** stimulated/activated B cells and antibody as a function of time for each group of disease severity from 10,000 independent simulations, error bars are shown as the standard deviation.

We attributed the level of disease severity to immune response kinetics that was dictated by the starting amount of lymphocytes. **Figures 7A–D** show the average number of lymphocytes for each disease-severity group, stimulated/activated B cells, and antibodies as a function of time after the second heterotypic infection. We noted that disease severity was strongly correlated with the starting amount of lymphocytes, including naïve B cells, CD4^+^ and CD8^+^ T cells (**Figure 7A**). Specifically, small amounts of all three lymphocyte types were associated with the severe-case phenotypes, whereas large amounts of all three lymphocyte types was associated with non-symptomatic cases. Secondly, delayed kinetics in the formation of stimulated B cells (**Figure 7B**) and activated B cells (**Figure 7C**) resulted in significantly slower production of antibodies (**Figure 7D**) for severe compared to non-symptomatic cases. The delay in the production of antibodies allows the virus to replicate and reach high viral concentrations among the severe cases.

Compared to lymphocytes, other immune factors exhibited weaker and indirect correlations with disease severity: secondary antibody response, immune recall, primary antibody response, and primary viral peak as shown in density plots **Figures S5A–D**. **Figure S5A** demonstrates a weakly inverse correlation (Pearson correlation coefficient *r*=-0.54) between peak-viral concentration in the secondary infection and the amount of antibodies generated in response to the secondary infection. In turn, **Figure S5B** shows a mild dependency (*r*=0.47) between the amount of antibodies generated in the secondary infection and the amount of immune recall viz-a-viz antibodies, long-lived plasma cells, and memory B cells generated from and retained for one year after the primary infection. Subsequently, **Figure S5C** exhibits a weakest link (*r*=0.22) between the amount of immune recall and the amount of antibodies generated during the primary infection. Finally, **Figure S5D** indicates a mildly reverse correlation (*r*=-0.45) between the amount of antibodies and peak viral concentration during the primary infection.

## Discussion

In this study, we extended our original affinity-maturation framework developed for malaria infections to model dengue infections. We made two major additions to account for the presence of T cells and viral replication process. The first addition allowed us to model the interactions between B cells and helper CD4^+^ T cells in response to the invasion by a dengue virus at the germinal center and the activation of cytotoxic CD8^+^ T cells for viral clearance. The second addition enables us to monitor viremia dynamics as the dengue virus undergoes replication during its growth period and its clearance by either antibodies or cytotoxic CD8^+^ T cells. The model was designed to have a minimal amount of adjustable parameters: the initial viral concentration, the number of virus serotypes, the number of epitopes per serotype, and the initial amount of lymphocytes as a range from a population of a certain age. To our knowledge, this work represents the first computational simulation of affinity maturation that incorporates both multiple serotypes and multiple epitopes, which are key requirements to modeling multiple dengue infections by various serotypes. We applied this approach to study the immune response to dengue infections of different serotypes and used the simulation results to recapitulate experimentally verifiable characteristics of the immune response in terms of specificity and cross-reactivity.

As a validation of the modeling strategy, our results showed that the computational model successfully captured observed key dynamic features of a typical dengue infection and immune response. The model recapitulated experimental data (19–21) with respect to the overall virus growth kinetics during any infection, in addition to specific aspects related to its lag time for incubation, growth rate, peak viremia range, and clearance rate. Also, our model showed similar signature trends of B cell dynamics and the activation process of B cells and T cells as clinical measurements (34).

For predicting outcome within the modeling framework, we performed simulations on multiple sets of ten thousand subjects with either homotypic or heterotypic infections. By classifying our simulation subjects into four different levels of disease severity based on the peak viral titer, we recapitulated dengue infection outcomes from clinical studies (31) as shown in **Table 1**. In this table, we quantified the percentage of subjects that fall into the four different disease categories using the separation values from k-means clustering. Our predictions of the frequency of clinical observations for heterotypic infections were within the estimated 95% confidence intervals for three categories: non-symptomatic, mild, and moderate. However, we under-predicted the frequency of severe cases (0.89%) by almost 2% based on the 95% confidence intervals (0.31%, 1.47%). The noted discrepancy can be attributed to many factors that are related to both the absolute idealization of any modeling method as well as the inherent ambiguity in data collection in a clinical setting. Although the absolute difference was not large enough to negate the modeling approach, it is a possible quantitative limitation. But we should note that the percentage of 5% reported by Salje et al. (31) for severe cases is at the high end of the 2–5% range of severe cases reported by other studies (40–43). In light of this, the difference between our prediction and clinical observations was minuscule. In contrast to heterotypic infections, our simulations of homotypic infections identified a large majority of the infected children (97.58%) as remaining non-asymptomatic (**Table 1**), while a small minority experienced mild or moderate symptoms of (2.42%). This agrees with a clinical study by Waggoner et al. (39) who observed that only one patient of out more than 3,500 participants aged 2–14 years experienced severe dengue symptoms; they also reported a small percentage of mild cases while the vast majority of cases were non-symptomatic.

Moreover, our model also allowed us to ask important questions probing the origin and progress of dengue infections. Comparison of non-symptomatic and severe cases from heterotypic infections demonstrated that overproduction of specific antibodies during primary infection induced a population of cross-reactive antibodies during secondary infection, which ultimately leads to severe disease manifestations. Because immune response is a non-linear process, a modest change in the input variables could result in major changes in rare events at the tail ends of the distribution of outcomes. Therefore, our stochastic model is particularly appropriate for simulating a distribution of outcomes. In contrast, deterministic models, which simulate the average outcome of a process, may not be able to capture rare events like severe dengue disease.

Furthermore, we attributed the level of disease severity to the starting amount of B and T lymphocytes that determined the immune response kinetics. On one hand, the low range of lymphocytes caused slow stimulation and activation of B cells, which led to a delayed antibody production that failed in hindering viral replication and triggered severe secondary infections. On the other hand, the high range of lymphocytes caused immediate stimulation and activation of B cells, which led to a responsive antibody production that succeeded in retarding viral replication and prompted only weak secondary infections. This agrees with a study by Green et al. (44) who performed blood analysis of 51 Thai children within 72 h of fever onset and with detectable plasma dengue viral RNA that found that absolute CD4^+^ T cell and CD8^+^ T cell counts were decreased in children with DHF compared with those with DF early in the course of illness.

In addition, secondary antibody response, immune recall, primary antibody response and primary viral peak exhibited somewhat weaker and indirect correlations with disease severity. Therefore, we drew two contrasting inferences about these factors and disease severity. On one hand, high amount of antibodies generated during the primary infection succeeded in reducing viral replication and contributed to a high amount of immune recall, which propelled production of a high amount of antibodies in order to reduce viral replication for the secondary infection, leading to weakened secondary infections as in the non-symptomatic cases. On the other hand, low amount of antibodies generated during the primary infection failed in reducing viral replication during the primary infection and contributed to a low amount of immune recall, which under-produced the amount of antibodies that failed to reduce viral replication during the secondary infection, leading to strengthened secondary infections as in severe cases.

Our study focused on only a narrow population of 6-12 years old children as being infected by dengue virus. These children belong to the most vulnerable group of being infected and they are often recommended as a targeted group for vaccination. Future studies should focus on a complete age range in order to examine the role of age on being infected and experiencing severe symptoms. These future studies should also model virus-specific immune responses by incorporating clinical data on virus-specific replication rate and stimulation of B/T cells into our model.

We developed the stochastic model of B cell affinity maturation to serve as a platform from which to carry out theoretical and experimental studies of the humoral and cell-mediated immunity. By modeling important cellular immune components, we can recapitulate dengue infection outcomes in addition to providing a detailed mechanistic characterization of the underlying shift from a protective primary antibody response to a non-protective secondary antibody response. We are currently exploring a number of directions in which to extend this model, such as the incorporation of both live-attenuated vaccine and inactivated vaccine in order to mimic vaccination studies and exploring different vaccination regimens. This would allow us to provide prediction of vaccine efficacy and a rationale for how to generate protective immune responses as a function of vaccine platforms and regimens. We hope that the work presented in this work highlights the potential for theoretical biology to investigate the mechanisms underlying experimental observations in immunology, and we will continue to explore the application of computational methods in advancing our understanding of basic immunological principles.

## Data Availability Statement

The raw data supporting the conclusions of this article will be made available by the authors, without undue reservation. The source code of our affinity maturation model is freely available on GitHub at https://github.com/hnguyenbhsai/immune-response-to-dengue.git.

## Author Contributions

HN developed the theoretical formalism with help from SC and AW. HN carried out the simulations and wrote the manuscript with support from SC, ATW, HF, FC, and AW. AW supervised the project. All authors contributed to the article and approved the submitted version.

## Funding

This work is supported by the U.S. Army Medical Research and Development Command under Contract No. W81XWH-20-C-0031 and the Military Infectious Disease Research Program.

## Author Disclaimer

The opinions and assertions contained herein are the private views of the authors and are not to be construed as official or as reflecting the views of the U.S. Army, the U.S. Department of Defense, or The Henry M. Jackson Foundation for the Advancement of Military Medicine, Inc. (HJF). This manuscript has been approved for public release with unlimited distribution.

## Conflict of Interest

The authors declare that the research was conducted in the absence of any commercial or financial relationships that could be construed as a potential conflict of interest.

## Publisher’s Note

All claims expressed in this article are solely those of the authors and do not necessarily represent those of their affiliated organizations, or those of the publisher, the editors and the reviewers. Any product that may be evaluated in this article, or claim that may be made by its manufacturer, is not guaranteed or endorsed by the publisher.
