# Supplementary material for: Stochastic Model of the Adaptive Immune Response Predicts Disease Severity and Captures Enhanced Cross-Reactivity in Natural Dengue Infections
Source: Front Immunol. 2021 Aug 17;12:696755. doi: 10.3389/fimmu.2021.696755 (PMC8416063; doi:10.3389/fimmu.2021.696755)

Stochastic model of the adaptive immune response predicts disease severity and captures enhanced cross-reactivity in natural dengue infections

Hung D. Nguyen[1,2]*, Sidhartha Chaudhury[3], Adam T. Waickman[4], Heather Friberg[5], Jeffrey R. Currier[5], Anders Wallqvist[1]*

[1]Biotechnology HPC Software Applications Institute, Telemedicine and Advanced Technology Research Center, U.S. Army Medical Research and Development Command, Fort Detrick, MD, United States;

[2]Henry M. Jackson Foundation for the Advancement of Military Medicine, Bethesda, MD, United States;

[3]Center for Enabling Capabilities, Walter Reed Army Institute of Research, Silver Spring, MD, United States;

[4]Department of Microbiology and Immunology, State University of New York Upstate Medical University, Syracuse, New York, United States of America

[5]Viral Diseases Branch, Walter Reed Army Institute of Research, Silver Spring, Maryland, United States of America.

*(HDN) hnguyen@bhsai.org

*(AW) sven.a.wallqvist.civ@mail.mil

Supplemental Data

Table S1: Sequences and parameter values for each epitope of each serotype

Serotype	Epitope	Sequence
1	PrM	aaaa aaaa aaaa aaaa aaaa
1	FL	bbbb bbbb bbbb bbbb bbbb
1	DIII	cccc cccc cccc cccc cccc
1	hinge	dddd dddd dddd dddd dddd
2	PrM	aaaa aaaa aaaa aaaa aaaa
2	FL	bbbb bbbb bbbb bbbb bbb**c**
2	DIII	cccc cccc cccc cccc **dddd**
2	Hinge	dddd dddd dddd ddd**a** **aaaa**
3	PrM	aaaa aaaa aaaa aaaa aaaa
3	FL	bbbb bbbb bbbb bbbb bbb**d**
3	DIII	cccc cccc cccc cccc **aaaa**
3	hinge	dddd dddd dddd ddd**b** **bbbb**
4	PrM	aaaa aaaa aaaa aaaa aaaa
4	FL	bbbb bbbb bbbb bbbb bbb**a**
4	DIII	cccc cccc cccc cccc **bbbb**
4	hinge	dddd dddd dddd ddd**c** **cccc**

Table S2: Parameter values for the immune system model

Model parameter	Symbol	Value
**Simulation conditions**		
Virus dose		10^4 copies/ml
**Virus parameters**		
Serotypes		4
Epitopes		4
Epitope PrM		
Immunogenicity	γ	0.85
Clearance	ρ	0.1
Antigenic distance		0
Epitope FL		
Immunogenicity	γ	1.50
Clearance	ρ	1.0
Antigenic distance		1
Epitope DIII		
Immunogenicity	γ	1.15
Clearance	ρ	1.0
Antigenic distance		4
Epitope hinge		
Immunogenicity	γ	1.50
Clearance	ρ	1.0
Antigenic distance		5
Virus formation rate	k_V	6.2 ml/(copies x d)
Virus decay rate	g_V	$(8.0\ h)^{-1}$
**B cell parameters**		
B cell enhancement factor	ε_B	10
Ab enhancement factor	ε_{Ab}	2.5
Naïve B cell formation rate*	k_N	$(R\ h)^{-1}$
Naïve B cell stimulation	σ_N	$(1\ d)^{-1}$
GC B cell stimulation (base)	σ_{base}	$(8\ h)^{-1}$
GC B cell stimulation (maximum)	σ_{max}	$(15\ min)^{-1}$
GC B cell replication rate	r	$(8\ h)^{-1}$
Mutation probability	μ	0.10
Differentiation probability	δ	0.10
Memory cell stimulation	σ_M	$(1\ d)^{-1}$

Ab production	k_{Ab}	1.0
Naïve B cell decay rate	g_N	$(4.5\ d)^{-1}$
GC B cell decay rate (base)	g_B	$(4.5\ d)^{-1}$
Plasma cell decay rate	g_P	$(3\ d)^{-1}$
Ab decay rate	g_{Ab}	$(10\ d)^{-1}$
**T cell parameters**		
$CD4^+$ T cell formation rate*	k_{T1}	R copies x $(ml\ x\ d)^{-1}$
$CD4^+$ T cell decay rate	k_{T2}	$(4.5\ d)^{-1}$
$CD8^+$ T cell formation rate*	k_{T3}	R copies x $(ml\ x\ d)^{-1}$
$CD8^+$ T cell decay rate	k_{T4}	$(4.5\ d)^{-1}$
$CD4^+$ T cell activation rate	k_{T5}	1200 ml x $(copies\ x\ h)^{-1}$
$CD4^+$ T cell differentiation rate	k_{T6}	$(15\ h)^{-1}$
Memory $CD4^+$ T cell formation rate	k_{T7}	$(15\ h)^{-1}$
Activated $CD4^+$ T cell decay rate	k_{T8}	$(4.5\ d)^{-1}$
Memory $CD4^+$ T cell reactivation rate	k_{T9}	2400 ml x $(copies\ x\ h)^{-1}$
$CD8^+$ T cell activation rate	k_{T10}	8 ml x $(copies\ x\ h)^{-1}$
$CD8^+$-based clearance rate	k_{T11}	0.000025 ml x $(copies\ x\ d)^{-1}$
$CD8^+$ T cell differentiation rate	k_{T12}	$(180\ h)^{-1}$
Activated $CD8^+$ T cell decay rate	k_{T13}	$(4.5\ d)^{-1}$

*R: the rate constant corresponds to either naïve B cell, CD4+, or CD8+ concentration that was randomly chosen for every simulation from a non-normal distribution of a healthy population of 6-12 years old children.

Figure S1: Simulation conditions for heterotypic and homotypic infections, each set is constructed from 10,000 independent simulations

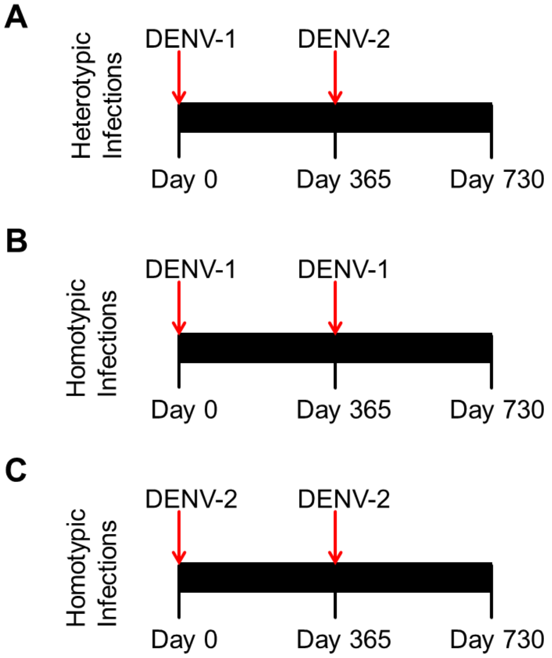

Figure S2: Affinity of germinal center B cells, memory B cells, plasma cells and antibodies from primary and secondary heterotypic infections as a function of time.

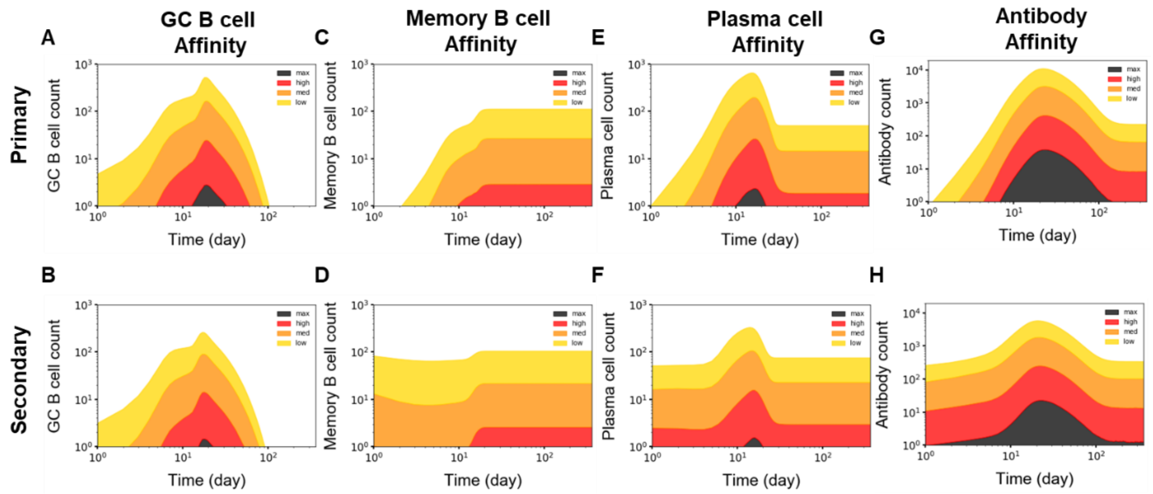

Figure S3: (A) Correlation between secondary viral peak and lymphocyte, (B) k-means clustering for disease classification, each of the four cluster is represented by a different color, the centroid of each cluster is represented as an orange dot. Each horizontal line represents a boundary value of secondary viral peak separating every two clusters.

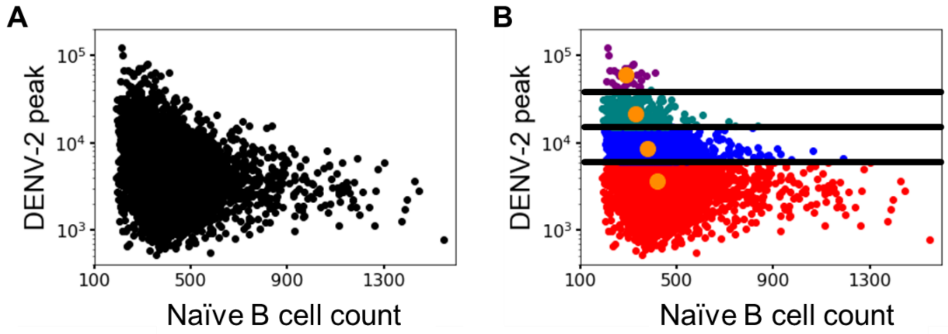

Figure S4: Profile of recalled memory, plasma, and antibody plus fresh naïve B cells before secondary infections

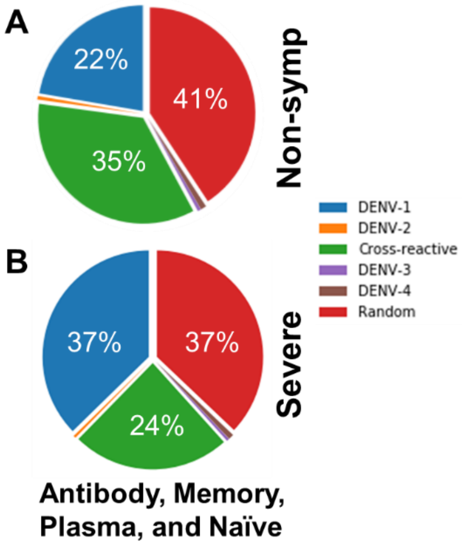

Figure S5: Correlation between disease severity (secondary viral peak), secondary antibody response, recalled memory, primary antibody response and primary viral peak. All data are plotted using log scales.

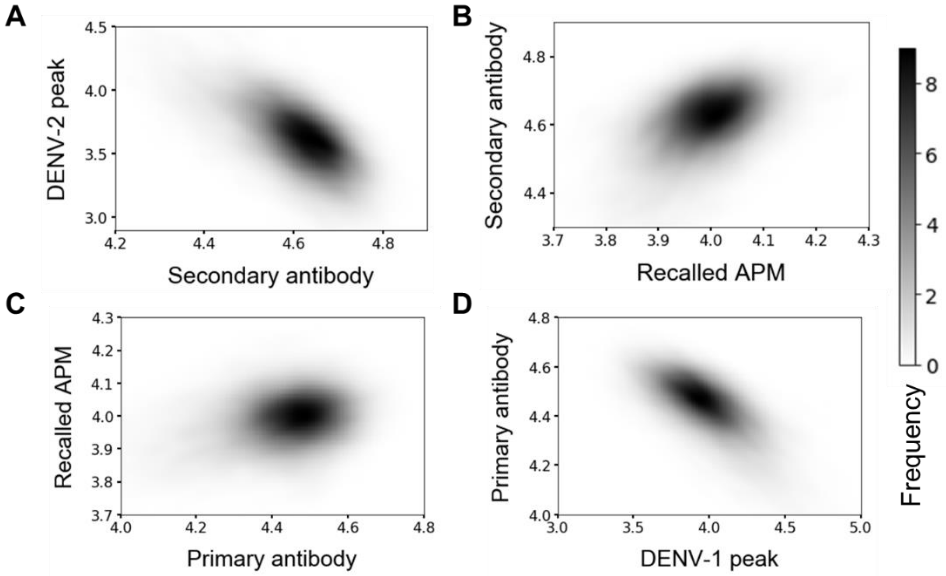

Supplement: Supplementary file 1 [file DataSheet_1.pdf]
